# A new approach to an old disease: Ageing, arrhythmogenic substrate and the role of risk‐factor modification in atrial fibrillation

**DOI:** 10.1113/EP093597

**Published:** 2026-07-11

**Authors:** Udit Thakur, Jonathan M. Kalman

**Affiliations:** ^1^ Department of Cardiology Royal Melbourne Hospital Melbourne Victoria Australia; ^2^ Department of Medicine University of Melbourne Melbourne Victoria Australia

**Keywords:** atrial fibrillation, atrial remodelling, risk factor modification

## Abstract

Atrial fibrillation (AF) prevalence rises sharply with age due to two independent mechanisms. Ageing produces irreversible arrhythmogenic substrate changes through progressive fibrosis, cellular senescence and mitochondrial dysfunction, while modifiable risk factors such as obesity, hypertension, sleep apnoea, diabetes and alcohol use promote atrial remodelling through inflammatory, haemodynamic and metabolic pathways. These comorbidities accumulate with age but operate through distinct mechanisms. Late gadolinium enhancement magnetic resonance imaging now quantifies fibrosis burden, while circulating biomarkers track inflammation, fibrosis and disease activity, and these measures respond to treatment, providing objective evidence of substrate modification. Landmark trials have transformed management. Weight loss of 10% or more when maintained is associated with reduced AF burden. Structured risk‐factor management may improve ablation outcomes and is associated with regression of AF type, with some patients moving from persistent back to paroxysmal patterns. These benefits occur across age groups, preventing arrhythmogenic substrate formation in younger patients and likely enabling reduction in AF burden in older adults. Collectively, this evidence positions risk‐factor management as the fourth pillar of AF care and shifts AF from an inevitable consequence of ageing to a modifiable chronic disease when delivered effectively.

## Abstract (highlights)

- **What is the topic of this review?**
  This review examines how atrial fibrillation arises from two interacting processes: irreversible age‐related change and reversible remodelling driven by obesity, hypertension, sleep apnoea, alcohol use and comorbidities.
- **What advances does it highlight?**
  Age‐related change and comorbidity‐driven remodelling act through distinct mechanisms, with the latter being potentially modifiable. Advances in imaging and biomarkers enable substrate quantification and monitoring of response to therapy. Clinical trials demonstrate that risk‐factor management improves AF outcomes, shifting care from symptom control toward disease modification.

## 1 INTRODUCTION

Atrial fibrillation (AF) remains the most prevalent sustained cardiac arrhythmia with major public health implications (Nattel et al., 2008). Though rare in youth, prevalence approaches 10% by 80 years, increasing morbidity, mortality and healthcare costs globally (Chen et al., 2018). Global AF and atrial flutter cases more than doubled between 1990 and 2019, from 28.3 to 60 million (Dong et al., 2023).

This increase reflects not only age‐related atrial changes but also systemic comorbidities and lifestyle factors (Chung et al., 2020). First, intrinsic age‐related changes occur within atrial tissue itself. These include progressive fibrosis, cellular senescence and electrical remodelling that create an increasingly arrhythmogenic substrate (Burstein & Nattel, 2008; Dun & Boyden, 2009). Second, and critically, modifiable comorbidities such as obesity, hypertension, heart failure, diabetes and obstructive sleep apnoea (OSA) independently promote atrial remodelling through distinct mechanisms (Lau et al., 2017; Medi et al., 2011; Sanders et al., 2003). While these comorbidities can affect patients at any age, their prevalence increases in older populations, creating a compound effect.

Understanding this dual mechanism has important therapeutic implications. While age‐related changes are largely irreversible, modifiable risk factors can be targeted therapeutically (Chung et al., 2020; Lau et al., 2017). Traditional AF management has relied on the principles of anticoagulation for stroke prevention, rate control for symptom management and rhythm control to maintain sinus rhythm (Kirchhof et al., 2016). These approaches, however, address consequences rather than causes. Accumulating clinical evidence indicates that targeting modifiable risk factors may result in reduction in AF burden (Abed et al., 2013). Risk factor optimization is increasingly recognized as the fourth pillar of contemporary AF care, representing a shift toward disease‐modifying rather than symptom‐directed therapy.

This review synthesizes current understanding of how ageing contributes to AF and how age‐related changes differ from comorbidity‐driven remodelling. We outline clinical tools to assess substrate and examine evidence supporting risk‐factor modification across all patients. This approach shifts AF management toward substrate‐directed, disease‐modifying care rather than symptom control.

## 2 MECHANISMS OF AGE‐RELATED ATRIAL REMODELLING

The development of an AF substrate involves complex structural, electrical and cellular changes within atrial tissue (Chen et al., 2018; Kistler et al., 2004). While these changes occur as part of normal ageing, they create the foundation upon which modifiable risk factors can further promote arrhythmogenesis (Lau et al., 2017). Understanding these mechanisms helps distinguish between what is inherently irreversible and what can be therapeutically targeted.

### 2.1 Structural changes: the architectural foundation of AF

Progressive atrial fibrosis is the hallmark of age‐related remodelling and a central driver of AF (Dun & Boyden, 2009). Ageing is the strongest non‐modifiable factor promoting atrial fibrogenesis, and autopsy studies show a clear increase in collagen content even in hearts without clinical disease (Burkauskiene, 2005; Horn & Trafford, 2016). This fibrotic tissue is not simply inert scar. Collagen deposition interrupts the normal alignment of atrial muscle bundles, forcing electrical impulses to take irregular routes that create zones of slow conduction and block (Morton et al., 2002). The resulting electrical heterogeneity provides the substrate for arrhythmia circuits. Fibroblasts potentially contribute to atrial remodelling through secretion of paracrine mediators, such as transforming growth factor‐β1 (TGF‐β1), connective tissue growth factor, platelet‐derived growth factor, endothelin‐1 and interleukin (IL)‐6. These factors have been shown to alter myocyte calcium handling, ion channel expression and conduction (Dzeshka et al., 2015). These changes may be reversible. In an ovine model of diet‐induced obesity, 30% weight reduction reversed atrial fibrosis, inflammation, endothelin‐B receptor upregulation and connexin‐43 loss with a trend toward TGF‐β1 reduction (*P* = 0.07). This indicates that these changes may be partially reversible with risk factor modification (Mahajan et al., 2021). The resulting heterogeneous fibrosis is a key determinant of arrhythmic risk, with mapping studies demonstrating progressive electroanatomical abnormalities that distinguish paroxysmal from persistent AF even in structurally normal hearts (Teh, Kistler et al., 2012).

Ageing also alters atrial structure through chamber dilation and cellular changes. Enlargement arises from both haemodynamic stretch and active remodelling, and larger atria can sustain more complex re‐entrant circuits, making AF more stable once initiated (Nattel et al., 2008). AF itself then promotes further dilation by impairing atrial contraction and increasing filling pressures (van de Vegte et al., 2021). At the myocyte level, ageing leads to gradual cell loss through apoptosis and necrosis, with surviving cells becoming hypertrophied and showing abnormal calcium handling that can trigger ectopic activity (Herraiz‐Martínez et al., 2015). Fatty infiltration contributes to a pro‐arrhythmic milieu through inflammatory and metabolic signalling that influences local conduction and structural remodelling (Dzeshka et al., 2015; Karam et al., 2017).

### 2.2 Electrophysiological changes: Disrupting electrical harmony

Ageing alters atrial electrophysiology in ways that create a fertile substrate for arrhythmia. Human mapping studies demonstrate a 15–20% reduction in conduction velocity from youth to old age, and this slowing is rarely uniform (Kistler et al., 2004). Non‐uniform progression of atrial remodelling creates intra‐atrial electrophysiological heterogeneity with marked regional variations in atrial conduction and refractoriness. Ageing also increases electrogram fractionation and dispersion of refractoriness across the atria (Kistler et al., 2004; Sanders et al., 2004). Similar changes occur within the pulmonary veins, where advancing age is associated with lower voltages, slower conduction and greater fractionation at the veno‐atrial junction (Teh, Kalman et al., 2012). This patchwork of electrophysiological change heightens the overall vulnerability of the aged atrium to arrhythmia. These alterations include shifts in ion‐channel behaviour, most notably a reduction in L‐type calcium current in ageing human atrial myocytes, which impairs calcium homeostasis and increases the likelihood of spontaneous calcium release and triggered activity (Herraiz‐Martínez et al., 2015).

### 2.3 Inflammatory and metabolic pathways

Inflammatory and metabolic disturbances play a central role in age‐related atrial remodelling. Mitochondrial dysfunction is a key early driver. Reduced ATP availability impairs calcium handling, while damaged mitochondria generate excess reactive oxygen species (ROS) (Pool et al., 2021). These ROS injure ryanodine receptors, promote spontaneous calcium leak, and activate pro‐fibrotic TGF‐β1 pathways (Xie et al., 2015).

Ageing is accompanied by a growing burden of senescent atrial myocytes, with human tissue studies showing clear increases in senescence markers in older individuals and in patients with atrial fibrillation (Adili et al., 2022). These cells exhibit a senescence‐associated secretory phenotype rich in inflammatory cytokines, matrix metalloproteinases and pro‐fibrotic mediators (Adili et al., 2022). This creates a self‐perpetuating environment in which senescent cells drive further senescence in neighbouring myocytes and fibroblasts (Admasu et al., 2021). Autophagy impairment provides an additional link between inflammation and fibrosis (Lin et al., 2020). Osteopontin, an inflammatory mediator upregulated several‐fold in atrial fibrillation, suppresses autophagy through phosphoinositide 3‐kinase/Akt signalling, reducing collagen turnover and allowing matrix to accumulate (Lin et al., 2020).

### 2.4 Mechanistic overlap

Age‐related and comorbidity‐driven pathways may not be mechanistically distinct. The same processes described above, including oxidative stress, inflammation, TGF‐β1 signalling and fibrosis, may potentially also drive atrial remodelling in obesity, hypertension and OSA (Karam et al., 2017; Lau et al., 2017). The distinction matters clinically because the comorbidity‐driven component of these shared pathways can be modified through intervention, whereas the age‐related component cannot.

## 3 IMAGING AND BIOMARKERS FOR SUBSTRATE QUANTIFICATION

Translating mechanistic insights into clinical practice requires tools to quantify the atrial substrate and distinguish irreversible age‐related changes from potentially reversible comorbidity‐driven remodelling. Table 1 provides a summary of commonly used imaging and biomarkers.

**TABLE 1:** Imaging and biomarkers for atrial substrate assessment.

Modality	Measure	Clinical signal	Interpretation
**LGE‐CMR**	Global and regional fibrosis	Higher fibrosis linked with higher recurrence (DECAAF)	Most specific tool for fibrosis assessment
**Echocardiography**	LA volume index and LA strain	LA volume index >34 mL/m^2^ and strain < 23% identify impaired function	Routine assessment of structure and function
**NT‐proBNP**	Wall stress	Strong predictor of incident AF and recurrence	Reflects haemodynamic load and active remodelling
**Galectin‐3**	Fibrosis marker	Associated with atrial enlargement and recurrence	Links molecular fibrosis to clinical phenotype
**CRP and IL‐6**	Inflammatory markers	Elevated levels reflect inflammatory remodelling	Provide insight into systemic inflammation
**GDF‐15**	Cellular stress marker	Reflects biological ageing	Differentiates ageing‐related substrate
**microRNAs**	miR‐21, miR‐29, miR‐328	Signals fibrosis and calcium‐handling pathways	Research markers of mechanistic change

Abbreviations: AF, atrial fibrillation; CRP, C‐reactive protein; GDF‐15, growth differentiation factor 15; IL‐6, interleukin 6; LA, left atrial; LGE‐CMR, late gadolinium enhancement cardiac magnetic resonance; NT‐proBNP, N‐terminal pro B type natriuretic peptide.

### 3.1 Advanced imaging of atrial fibrosis

Late gadolinium enhancement cardiac magnetic resonance (LGE‐CMR) has become the leading non‐invasive technique for characterizing atrial fibrosis. The landmark DECAAF study demonstrated its clinical relevance in 260 patients undergoing first‐time ablation, showing a clear dose–response relationship between fibrosis burden and recurrence (Marrouche et al., 2014). Patients with minimal enhancement (<10%) had a 14% recurrence rate at 1 year, compared with 43% for moderate fibrosis (10–20%) and 75% for extensive fibrosis (>30%) (Marrouche et al., 2014). These gradients have since been reproduced in multiple cohorts and continue to inform patient selection and procedural planning (Siebermair et al., 2017). Beyond global fibrosis burden, improvements in acquisition and post‐processing now allow visualization of regional enhancement patterns. Early studies suggest that these maps may help identify arrhythmogenic zones or regions prone to incomplete lesion formation (Siebermair et al., 2017). Although still developing, these techniques offer a potential bridge between mechanistic insights and procedural strategy.

Echocardiography remains the clinical standard. A left atrial volume index above 34 mL/m^2^ identifies patients at heightened risk of AF, while reduced atrial reservoir strain (<23%) provides incremental information on atrial dysfunction independent of size (Tomlinson et al., 2021). Together, these measures offer a practical means of assessing the atrial substrate in routine care and complement the more detailed structural information available from CMR.

### 3.2 Biomarkers

Biomarkers have the potential to complement imaging for substrate assessment, offering molecular insight into atrial stress, inflammation and fibrosis. However, as yet this potential remains largely unrealized, and none are currently incorporated into standard AF management algorithms. N‐terminal pro B type natriuretic peptide (NT‐proBNP) is the most consistently validated biomarker in AF (Wang et al., 2025). It reflects atrial pressure and wall stress and is strongly associated with incident AF, progression from paroxysmal to persistent forms, and recurrence after cardioversion or ablation (Blessberger et al., 2022). Levels rise with both ageing‐related structural change and modifiable disease activity, making it one of the most robust circulating markers of atrial remodelling (Wang et al., 2025). Galectin‐3 is another biomarker associated with atrial fibrosis and the arrhythmogenic substrate in AF (da Silveira et al., 2020). Elevated levels correlate with atrial enlargement and predict recurrence after ablation, although data linking it directly to quantified fibrosis burden remain evolving (da Silveira et al., 2020).

Beyond these established markers, several emerging biomarkers offer mechanistic signals but remain research focused. Growth differentiation factor‐15 (GDF‐15) reflects cellular stress and biological ageing, while C‐reactive protein (CRP) and IL‐6 capture inflammatory components of remodelling (Hijazi et al., 2016). MicroRNA signatures such as miR‐21, miR‐29 and miR‐328 offer molecular insight into fibrosis and calcium‐handling pathways, although their clinical role remains exploratory (Luo et al., 2015).

### 3.3 Clinical scores for substrate prediction

Clinical risk scores provide a practical way to estimate atrial substrate before invasive assessment. Tools such as the APPLE and DR‐FLASH scores combine age, AF type and structural or metabolic features to identify patients more likely to harbour low‐voltage areas on electroanatomic mapping (Kornej et al., 2018). While these scores do not replace imaging or biomarkers, they offer a simple method to identify individuals with potentially advanced remodelling who may require broader substrate directed strategies.

Imaging, biomarkers and clinical scores offer complementary insights into the atrial substrate. Imaging defines anatomy, biomarkers capture molecular activity, and clinical scores provide a practical estimate of remodelling severity. Together, they enable more precise patient stratification, though integrating them into routine practice remains an evolving challenge.

## 4 RISK FACTOR MANAGEMENT AS A REMODELLING MODIFIER

Understanding that atrial remodelling reflects both ageing and modifiable disease activity has shifted AF care toward a new way of thinking about an age‐old disease. Structured risk factor management now stands as a practical means of reducing AF burden and altering the course of the condition.

**FIGURE 1:**
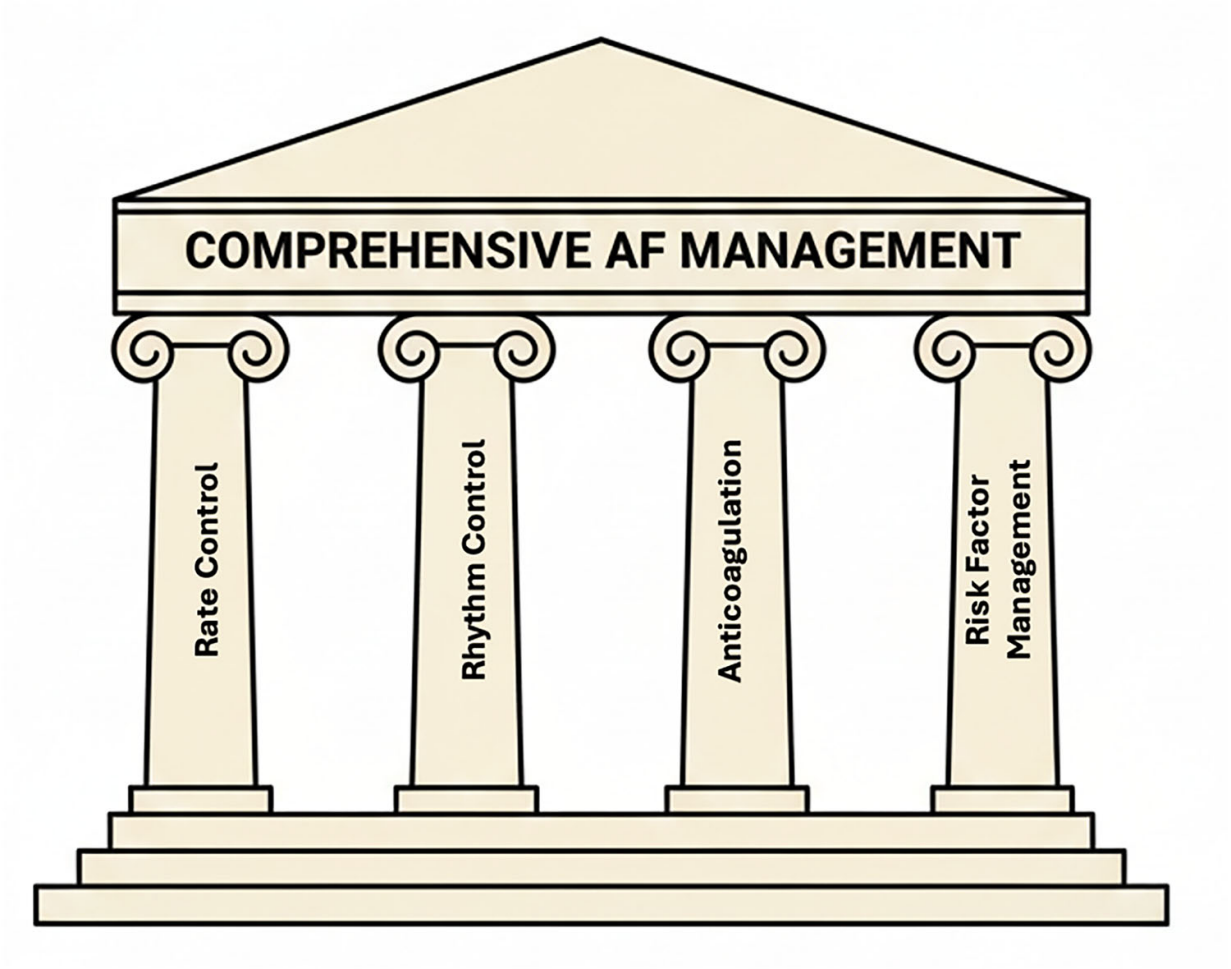
Four pillars of atrial fibrillation management. Illustration of the four pillars of atrial fibrillation management: anticoagulation, rate control, rhythm control and structured risk‐factor management. This figure has been reproduced and modified with permission from Lau et al. (2017). Modifiable risk factors and atrial fibrillation. *Circulation* 136, 583–596.

Risk factor modification now sits alongside conventional AF therapies as the fourth pillar of management (Figure 1) (Kirchhof et al., 2016; Van Gelder et al., 2024). Standard approaches control symptoms or prevent complications but do not change the substrate (Nattel et al., 2008). The newer framework recognizes AF as a systemic condition shaped by obesity, hypertension, OSA, diabetes, alcohol excess and low physical activity (Chung et al., 2020). These factors are not simple associations. They promote inflammation, oxidative stress, fibrosis and electrical instability within the atria and accelerate age‐related remodelling (Chung et al., 2020; Lau et al., 2017). Addressing them in a structured and sustained way can reduce the burden of disease activity within atrial tissue and offers a realistic opportunity to slow or reverse progression (Abed et al., 2013).

## 5 EVIDENCE FROM CLINICAL TRIALS

### 5.1 Establishing causality

Abed et al. (2013) provided the first randomized evidence that structured weight loss can modify the AF burden. In 150 obese patients with symptomatic AF randomized to intensive weight management and cardiometabolic risk factor optimization versus general lifestyle advice, the intervention group showed clear reductions in both symptom burden and AF episodes. Patients who achieved at least 10% weight loss showed the greatest improvements, including reductions in left atrial volume, decreases in left ventricular mass and favourable changes in metabolic profile. These findings established causality for AF burden reduction through risk factor management and demonstrated that obesity‐related drivers of remodelling, including inflammation, atrial stretch and metabolic dysfunction, can improve with sustained intervention.

### 5.2 Long‐term outcomes: The LEGACY study

The LEGACY study provided definitive long‐term evidence that sustained weight loss can modify the natural history of AF (Pathak et al., 2015). In a cohort of 355 overweight patients followed for 5 years, a clear dose–response relationship emerged. Twenty percent of those who lost less than 3% of body weight achieved freedom from AF, compared with 35% of those who lost between 3% and 9%, and 86% of those who achieved at least 10% loss. Weight stability proved essential. Fluctuations greater than 5% doubled the risk of recurrence, highlighting that sustained change rather than short‐term reduction drives long‐term benefit. Patients who maintained at least 10% loss continued to improve throughout follow up, whereas those with fluctuating weight often experienced late recurrence despite early gains. This pattern supports genuine modification of the AF trajectory rather than temporary suppression.

### 5.3 Enhancing ablation outcomes: ARREST‐AF cohort

The ARREST‐AF cohort study evaluated structured risk factor management in 149 patients with AF and a body mass index above 27 kg/m^2^ undergoing catheter ablation (Pathak et al., 2014). The programme was comprehensive and physician led, with targets that included at least 10% weight loss, blood pressure below 130/80 mmHg, improved glycaemic and lipid control, treatment of OSA, smoking cessation and reduced alcohol intake. Over a median 42 months of follow‐up, arrhythmia‐free survival was 42% in the risk factor management group compared with 13% in controls without antiarrhythmic drugs. Participation in the programme remained the strongest predictor of success with a hazard ratio of 4.8, exceeding AF type, AF duration, left atrial size and procedural characteristics. These findings showed that structured risk factor management improves ablation outcomes by addressing the underlying drivers of AF rather than simply supporting the procedure.

### 5.4 Single factor intervention: The Alcohol‐AF trial

The Alcohol‐AF trial demonstrated that modifying a single lifestyle factor can influence AF outcomes (Voskoboinik et al., 2020). In 140 regular drinkers with paroxysmal or persistent AF randomized to abstinence or continued intake, abstinence reduced recurrence from 73% to 53% and lengthened time to first recurrence (hazard ratio 0.55). AF burden over 6 months was lower in the abstinence group, with a median burden of 0.5% compared with 1.2% in controls. Alcohol intake fell by 87.5% in the abstinence group and by 19.5% in controls. These effects were accompanied by modest weight loss, fewer AF‐related admissions and better symptom scores. Mechanistically, alcohol promotes autonomic instability, acute inflammation, atrial ectopy and atrial stretch, and reducing exposure lowers disease activity independent of weight change (Voskoboinik et al., 2020).

### 5.5 Reversing disease trajectory: REVERSE‐AF

The REVERSE‐AF study showed that AF progression is not inevitable and, in some cases, may regress with sustained intervention (Middeldorp et al., 2018). Among 355 overweight patients offered structured weight and risk factor management, those who achieved sustained control demonstrated clear regression of disease. In the highest weight loss group (≥10%), only 3% progressed from paroxysmal to persistent AF, whereas 88% with persistent AF regressed to paroxysmal AF or no AF in the final year of follow‐up. In contrast, 41% progressed in the lowest weight loss group and only 1% regressed. Follow‐up averaged around 48 months across groups. These findings show that AF trajectory is shaped by modifiable disease activity rather than ageing alone, and that AF trajectory remains modifiable even after persistent AF develops.

### 5.6 Obstructive sleep apnoea as a modifiable contributor

OSA promotes atrial remodelling through intermittent hypoxia, negative intrathoracic pressure swings and autonomic instability (Nalliah et al., 2022). The SLEEP‐AF study showed that this substrate is at least partly reversible (Nalliah et al., 2022). Patients with AF and moderate‐to‐severe OSA treated with continuous positive airway pressure (CPAP) demonstrated faster conduction velocity, higher atrial voltages and less electrogram fractionation than controls. While CPAP clearly improves the underlying substrate, evidence that it prevents AF recurrence as a standalone therapy is less consistent, and its greatest value appears to be as an adjunct alongside ablation and broader risk factor management (Chung et al., 2020).

### 5.7 Integrated evidence for substrate modification

Across trials, risk‐factor modification produces measurable changes in atrial structure, loading and electrophysiological behaviour (Table 2). Intensive weight loss programmes in Abed et al. and LEGACY reduced left atrial size and septal thickness on echocardiography, accompanied by marked improvements in blood pressure, glycaemic control, lipid profile and metabolic markers (Abed et al., 2013; Pathak et al., 2015). REVERSE‐AF demonstrated a clear dose response effect, with greater weight loss associated with progressive reduction in left atrial dimensions and substantial regression of AF type over 4 years of follow up (Middeldorp et al., 2018). Alcohol‐AF confirmed that removing a single exposure can reduce AF burden and prolong time to recurrence, highlighting a rapid and reversible impact on atrial excitability and arrhythmia triggers (Voskoboinik et al., 2020). The ARREST‐AF cohort study showed that a structured physician‐led programme targeting weight (≥10% loss), blood pressure (<130/80 mmHg), glycaemia (HbA1c ≤6.5%), lipids (low‐density lipoprotein <100 mg/dL), OSA, smoking and alcohol intake improves cardiometabolic indices and supports sustained sinus rhythm after ablation, reinforcing the relationship between metabolic and haemodynamic improvement and substrate stability (Pathak et al., 2014).

**TABLE 2:** Major trials of risk factor management in AF.

Trial	Design (sample size)	Population and intervention	Key outcome
Abed et al. (2013)	RCT (*n* = 150)	Obese symptomatic AF patients receiving structured weight and cardiometabolic management	Patients achieving at least 10% weight loss had clear reductions in AF symptoms and AF episodes, along with favourable reductions in left atrial size and ventricular mass
**LEGACY**	Prospective cohort (*n* = 355)	Overweight AF cohort with long‐term weight tracking	Sustained weight loss of at least 10% was associated with 86% freedom from AF, while weight fluctuation increased recurrence risk
**ARREST‐AF (cohort)**	Prospective cohort (*n* = 149)	AF patients with BMI above 27 undergoing catheter ablation and a structured programme	Arrhythmia‐free survival was 42% compared with 13% in usual care, showing improved ablation outcomes with risk‐factor optimization
**Alcohol‐AF**	RCT (*n* = 140)	Regular drinkers with AF randomized to abstinence or continued alcohol intake	Abstinence reduced AF recurrence from 73% to 53%, lowered AF burden and improved symptoms
**REVERSE‐AF**	Prospective cohort (*n* = 355)	Overweight AF cohort undertaking structured weight and risk‐factor management	In patients achieving at least 10% weight loss, only 3% progressed, and 88% regressed from persistent AF, indicating regression of disease trajectory
**SLEEP‐AF**	RCT (*n* = 24)	AF patients with moderate to severe OSA randomized to CPAP versus usual care	CPAP reversed substrate remodelling, improving conduction velocity, voltage and electrogram fractionation compared to controls

Abbreviations: AF, atrial fibrillation; BMI, body mass index; CPAP, continuous positive airway pressure; OSA, obstructive sleep apnoea; RCT, randomized controlled trial; RFM, risk factor management.

### 5.8 Biological mechanisms linking risk factor modification to substrate change

These clinical benefits reflect attenuation of pathophysiological pathways that are distinct from intrinsic ageing. Weight loss reduces epicardial adipose tissue volume and lowers secretion of pro‐inflammatory and pro‐fibrotic mediators including IL‐1β, IL‐6, Activin‐A and tumour necrosis factor‐α (Al‐Kaisey & Kalman, 2021). This environment favours collagen turnover and limits new fibrosis formation, changes that align with the structural improvements seen in LEGACY and REVERSE AF (Middeldorp et al., 2018; Pathak et al., 2015). Haemodynamic gains from reduced blood pressure, improved ventricular compliance and treatment of OSA lower atrial wall stress and prevent stretch‐mediated electrical instability (Chung et al., 2020). Metabolic improvements in insulin sensitivity reduce lipotoxic and oxidative stress within atrial myocytes (Karam et al., 2017; Pathak et al., 2014). Exercise‐related increases in fitness enhance mitochondrial function, improve endothelial performance and restore autonomic balance (Kim et al., 2023).

**FIGURE 2:**
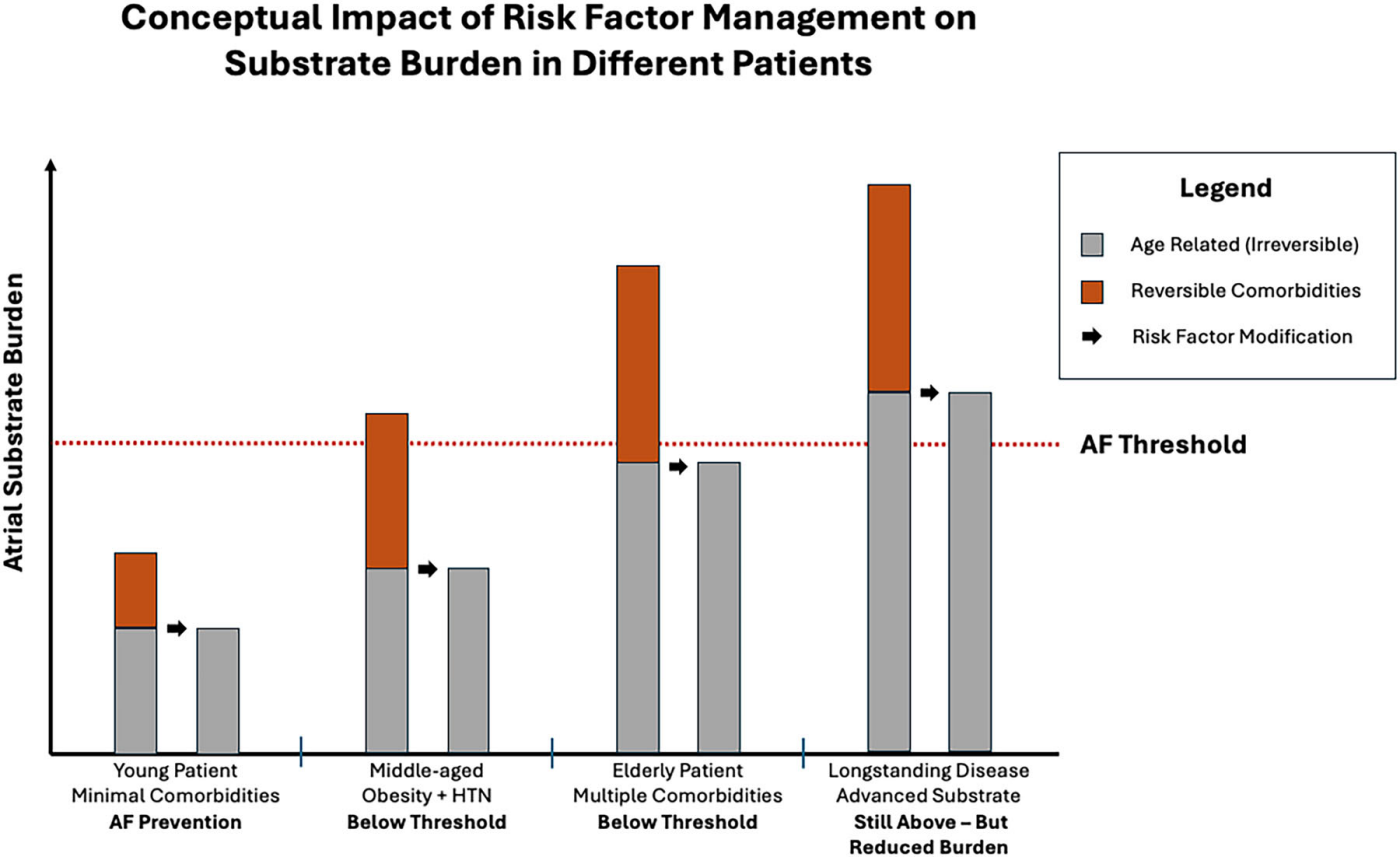
Effect of risk‐factor management across age groups. Conceptual illustration of how ageing and modifiable risk factors contribute to atrial substrate development. The diagram depicts the hypothesized interaction between age‐related structural change and modifiable metabolic or inflammatory drivers, acknowledging that the relative contribution of each component varies substantially between patients. HTN, hypertension.

The critical distinction is that these mechanisms target active and modifiable drivers rather than irreversible age‐related change. Cellular senescence and age‐associated fibrosis may persist, but the inflammatory, haemodynamic and metabolic contributors to atrial instability respond rapidly and often robustly to intervention (Dun & Boyden, 2009; Horn & Trafford, 2016; Middeldorp et al., 2018). This explains the consistency of benefit across age groups (Abed et al., 2013; Pathak et al., 2015). Younger patients suppress early formation of substrate, while older patients with established disease still achieve meaningful improvement by reducing the load of modifiable stressors (Figure 2). Once these pathways are attenuated, many patients fall below the threshold needed to sustain AF, despite the presence of fixed age‐related changes (Middeldorp et al., 2018; Pathak et al., 2015).

### 5.9 Risk factor modification complements rhythm control

Risk factor management should not be viewed in isolation from rhythm control but rather as part of the broader rhythm control strategy alongside antiarrhythmic medications and catheter ablation. The EAST‐AFNET 4 trial showed that early rhythm control within 1 year of diagnosis reduces adverse cardiovascular outcomes (Kirchhof et al., 2020), and the 2024 ESC guidelines now recommend early and sustained rhythm control as a central goal (Van Gelder et al., 2024). Within this framework, medications and ablation address arrhythmia control directly, while risk factor management targets the upstream drivers of substrate formation. The ARREST‐AF experience illustrates this directly, with structured risk factor management markedly improving arrhythmia‐free survival after ablation (Pathak et al., 2014). For many patients, optimal rhythm control may require integration of all these approaches.

### 5.10 Trial limitations

Several limitations of the existing evidence base warrant consideration. Much trial data supporting risk factor management in AF originates from observational cohorts at a single centre with dedicated multidisciplinary infrastructure (Middeldorp et al., 2018; Pathak et al., 2014, 2015). Patients achieving the greatest weight loss were inherently self‐selected, and the dose–response relationship between weight loss and AF freedom may partly reflect adherence bias. Abed et al. (2013) from the same group demonstrated reduction in AF symptom burden in a randomized trial of weight loss. The Alcohol‐AF trial was a multicentre randomized study demonstrating significant reduction in AF burden with alcohol abstinence. The trial was nevertheless relatively small (sample size 140), needed to screen over 750 patients to reach targeted enrolment of 140, limiting generalizability, and follow‐up duration was 6 months only. The short‐term follow‐up occurred as many patients in the abstinence arm were not prepared to continue for a longer duration (Voskoboinik et al., 2020).

Clinical trials have demonstrated reductions in AF burden, LA size and AF type regression, but direct evidence of substrate reversal in humans is largely limited to preclinical models (Mahajan et al., 2021). No AF risk factor trial has measured fibroblast‐derived mediators or autophagy biomarkers longitudinally, and high‐sensitivity (hs)‐CRP (Abed et al., 2013) remains the only inflammatory marker reported before and after intervention.

## 6 FUTURE DIRECTIONS

The heterogeneity of atrial fibrillation demands more precise phenotyping to guide treatment selection. A multi‐modal approach that integrates LGE‐CMR for fibrosis burden, biomarkers for disease activity and structured risk factor profiling will allow predictive models to identify which patients derive the greatest benefit from specific interventions (Al‐Kaisey & Kalman, 2021; Chung et al., 2020; Luetkens et al., 2018). Younger patients with low fibrosis but multiple modifiable drivers may achieve sustained reduction in AF burden with lifestyle and medical optimization alone, whereas older patients with moderate substrate disease are more likely to require a combination of risk‐factor management and interventional therapy such as catheter ablation (Chung et al., 2020; Pathak et al., 2014). A precision medicine framework built on these features will help distinguish those who respond to risk factor management from those who need earlier invasive therapy.

Implementation remains the critical barrier. Traditional electrophysiology clinics lack infrastructure for sustained lifestyle support. Success requires dedicated multidisciplinary AF risk‐factor clinics, as demonstrated in ARREST‐AF, where physicians, dietitians, exercise physiologists and behavioural specialists worked collaboratively to deliver structured lifestyle and medical optimization (Pathak et al., 2014). Digital health platforms show promise for younger patients while older populations benefit from in‐person group support (Linz et al., 2024). Centres that have implemented structured multidisciplinary programmes report strong engagement over long‐term follow‐up, with patients attending regular 3‐monthly reviews in dedicated lifestyle clinics (Middeldorp et al., 2018; Pathak et al., 2014, 2015).

Glucagon‐like peptide‐1 (GLP‐1) receptor agonists represent an emerging pharmacological option that may complement lifestyle‐based risk factor management. These agents produce sustained weight loss, improve metabolic and inflammatory profiles and therefore reduce upstream drivers of atrial remodelling (Saglietto et al., 2024). A recent systematic review and meta‐analysis reported a meaningful reduction in incident atrial fibrillation compared with placebo in high cardiovascular risk populations, with effects consistent across drug formulations and not limited to patients with diabetes (Saglietto et al., 2024). The AF data for GLP‐1 receptor agonists remain indirect, drawn from cardiovascular outcome trials in high‐risk populations rather than dedicated AF studies. The metabolic and anti‐inflammatory profile of these agents fits well with the pathways targeted by risk factor management, but dedicated AF trials are needed before they can be positioned as part of routine AF care.

Novel therapeutics may eventually address currently irreversible substrate. Agents targeting cellular senescence show early promise in preclinical and early clinical trials, though translation to AF requires validation (Yang et al., 2025). Anti‐fibrotic agents targeting TGF‐β pathways could complement lifestyle interventions (Chen et al., 2025). Future regenerative approaches using stem cells or gene editing remain experimental but offer hope for reversing age‐related changes (Yoo et al., 2021). Until then, aggressive risk factor modification remains our most powerful tool for reducing AF burden and modifying disease trajectory across all patient populations.

This emerging framework fundamentally reimagines an old disease. For decades, AF was treated as an electrical disorder requiring electrical solutions: drugs to suppress firing, ablation to eliminate triggers and cardioversion to reset rhythm. It is now clear that AF is a systemic condition in which the atrium functions as an end organ shaped by broader cardiometabolic health. This mirrors shifts in other chronic diseases where upstream prevention has replaced downstream damage control. Just as coronary disease is addressed before myocardial infarction, atrial disease should be targeted before persistent AF develops. The future of AF management lies in identifying and modifying substrate early, transforming a long‐standing arrhythmia into a preventable condition through systematic application of the fourth pillar across at‐risk populations.

## 7 CONCLUSION

Atrial fibrillation‐associated remodelling reflects a combination of fixed age‐related change and potentially reversible changes driven by obesity, hypertension, OSA, alcohol use and metabolic dysfunction. Accumulating evidence indicates that structured risk‐factor management, positioned as complementary to standard rhythm therapies, may improve AF outcomes across all ages. The challenge ahead lies in implementation: translating evidence gained under clinical trial conditions into routine practice through multidisciplinary clinics and scalable delivery models.

## AUTHOR CONTRIBUTIONS

This work was conducted at the Department of Cardiology, Royal Melbourne Hospital, Melbourne, Australia. Udit Thakur and Jonathan M. Kalman contributed to the conception and design of the work. Both authors drafted the manuscript and collectively revised it critically for important intellectual content. Both authors approved the final version of the manuscript, agree to be accountable for all aspects of the work in ensuring that questions related to the accuracy or integrity of any part of the work are appropriately investigated and resolved. All persons designated as authors qualify for authorship and all those who qualify are listed.

## CONFLICT OF INTEREST

None.

## GENERATIVE AI STATEMENT

No generative AI tools were used in the preparation of this manuscript.
